# Reducing pain in acute herpes zoster with plain occlusive dressings: a case report

**DOI:** 10.1186/s13256-015-0560-5

**Published:** 2015-04-25

**Authors:** David A Keegan

**Affiliations:** University of Calgary, G329, 3330 Hospital Drive NW, Calgary, AB T2N 4N1 Canada

## Abstract

**Introduction:**

The pain of acute herpes zoster (shingles) is severe and difficult to control. The medications used to control pain have a variety of important and potentially serious side effects. To the best of my knowledge, this is the first case report of using a plain topical occlusive dressing to reduce the pain of herpes zoster, avoiding the use of medication.

**Case presentation:**

A 40-year-old Caucasian man and a qualified physician (the author), developed a dermatomal vesicular rash consistent with herpes zoster. Applying plain topical occlusive dressings reduced the severity of his pain to an ignorable level.

**Conclusion:**

Plain topical occlusive dressings provide effective pain relief for acute herpes zoster, thereby avoiding the risks accompanying medication use.

## Introduction

I describe the case of a patient (the author) with acute herpes zoster (shingles), treated using a plain topical occlusive dressing to reduce the pain, thereby avoiding the use of medication. The lifetime risk of acquiring herpes zoster (for unvaccinated individuals) has been estimated to be 32% for women and 22% for men [1]. The acute pain of herpes zoster can be debilitating [2], and satisfactory pain control is difficult to achieve [2]. Patients can have a varied pain presentation including severe pain, hypersensitivity, and allodynia (pain in response to normally non-painful stimuli) [3]. Currently recommended therapies for acute severe pain include non-steroidal anti-inflammatories, narcotics, prednisone, and gabapentin [2], all of which carry risks of a variety of severe side effects and complications.

Some studies have evaluated the use of topical analgesia for the acute pain of herpes zoster. One uncontrolled study of lidocaine in petroleum jelly suggested a role for this therapy, but noted there were a large number of practical factors in applying the dressings and keeping them on that limited its usefulness [4]. Another study was a blinded comparison between patients who received aqueous patches containing lidocaine and other patients who received plain aqueous patches [5]. In this study, while there was some pain score improvement in the patients who received the plain aqueous patches, there was a more substantial improvement in those who received the lidocaine aqueous patches [5].

## Case presentation

The author, a 40-year-old Caucasian man and a qualified physician, developed a ‘barbed-wire-like’ painful left lateral thigh vesicular eruption in a dermatomal pattern. The rash had been preceded by two days of ‘electric, aching’ pain without skin changes at the same site, and occurred during a period of significantly reduced sleep due to an unexpected death of a sibling. The suspected diagnosis of herpes zoster was confirmed within four hours of the vesicle eruption, at an urgent care centre, and a seven-day course of oral valacyclovir 1000mg, administered orally three times a day (GlaxoSmithKline, Mississauga, Canada), was started immediately, along with naproxen (Bayer Inc., Mississauga, Canada) and oral codeine (Ratiopharm, Toronto, Canada) for analgesia.

The codeine and naproxen had minimal impact on the severity of his pain, which was constantly present. His pain was further aggravated by any contact with the affected skin, including contact by clothing. Altogether, this pain constellation led to poor sleep, difficulty in concentrating, and increased stress during an already stressful period.

**Figure 1:**
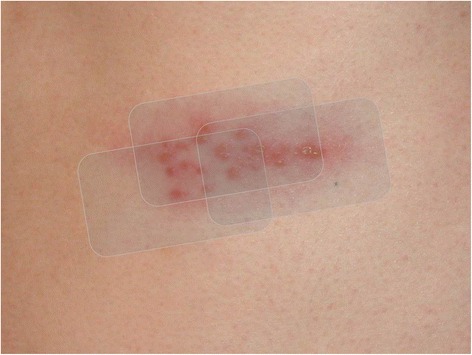
Depiction of occlusive dressings over vesicular lesions. Alteration of Creative Commons Licensed work by Preston Hunt; original photograph accessible at http://commons.wikimedia.org/wiki/File:Shingles_on_the_chest.jpg.

In an effort to avoid increased narcotic use or additional medications (and their possible side effects), he elected to apply a series of plain occlusive dressings (Tegaderm™; 3M, London, Canada) directly over the vesicular eruption, as depicted in Figure 1. This decision was based upon a spontaneous hypothesis that an additional layer of skin (albeit artificial) might reduce the severity of his constant pain and skin hypersensitivity.

His pain reduced immediately to a tolerable (and ignorable) level, from approximately eight out of 10 (with codeine and naproxen) to four out of 10 on a numerical pain scale (with 0 being no pain and 10 being severe pain). More importantly, incidental contact by clothes and water in the shower no longer resulted in additional severe lancing pain. Subsequent to the occlusive dressing application, no more analgesics were taken, and his pain level never increased above four out of 10 on the numerical pain scale. The occlusive dressings were applied for the duration of the vesicles, being finally removed after eight days. Peeling dressings were removed and replaced during this period. The removal of partially peeling dressings resulted in mild worsened pain at the time of removal, akin to removing a regular adhesive bandage. If replacement was delayed for longer than five minutes, his previous severe level of pain would begin to return, and promptly returned to four out of 10 or lower with subsequent reapplication. Full resolution of his symptoms occurred over approximately two and a half weeks, with no post-herpetic neuralgia.

## Discussion

The intervention described here is an attractive option as it removed the need for oral analgesia for the constant pain and hypersensitivity caused by herpes zoster. As a result, all potential side effects and potential drug interactions of oral medications were avoided. The occlusive dressing reduced his constant pain by half and greatly limited his hypersensitivity, bringing it to a tolerable level, allowing resumption of regular activities. It is possible that these occlusive dressings may work for other pain variants of acute herpes zoster, though this should be studied.

It has been previously proposed that herpes simplex virus (HSV) lesions may be treated with plain occlusive dressings, based upon a histological analysis that suggested HSV skin injury is similar to partial-thickness skin wounds [6]. The same rationale has been used, by extension, to support occlusive dressing use in the care of herpes zoster eruptions [7].

A PubMed search did not reveal any clinical therapeutic studies of any type evaluating the use of plain occlusive dressings for analgesia in this condition. However, in a blinded control study by Lin *et al*. [5], the control arm was an aqueous (non-active) patch. The substantial pain relief by the plain Tegaderm™ in this case report was more in keeping with the better pain relief experienced by patients in the aqueous lidocaine arm of the study by Lin *et al*. [5]. It is possible that while the patch itself protected the skin, the aqueous base aggravated the inflamed skin, thereby limiting the potential benefit of the physical barrier of the patch. More study needs to be done to see if the thin, plain Tegaderm™ dressings are equal in analgesia to lidocaine aqueous patches.

A reasonable clinical management approach might be to recommend plain Tegaderm™ use (as was done here) and ensure patients have multiple spare dressings, so as to allow removal and reapplication when applied dressings become problematic (as a result of peeling, bunching, or water interposition). The potential reduction in acute pain severity this option provides (without the complications of traditional analgesia) to patients with herpes zoster is large, given the difficulty in achieving satisfactory pain control with usual therapy.

## Conclusion

Plain occlusive dressings may provide effective analgesia for acute herpes zoster, thereby avoiding the risks accompanying medication use.

## Patient perspective

Herpes zoster was most unwelcome in the days immediately following my sister’s unexpected death. It was a great relief that I had an instant reduction of pain after applying the Tegaderm™ dressings, as it meant I could then focus on the needs of my family and issues related to her estate. It was months later that I fully realized this was an important clinical finding to share.

## Consent

The patient described in the case and the author of the case are the one and same individual (me). By submitting this case report, I am consenting for this case to be published.
